# Enhanced recovery after surgery (ERAS) in cytoreductive surgery (CRS) and hyperthermic intraperitoneal chemotherapy (HIPEC): a cross-sectional survey

**DOI:** 10.1515/pp-2021-0117

**Published:** 2021-06-21

**Authors:** Geetu Bhandoria, Sohan Lal Solanki, Mrugank Bhavsar, Kalpana Balakrishnan, Cherukuri Bapuji, Nitin Bhorkar, Prashant Bhandarkar, Sameer Bhosale, Jigeeshu V. Divatia, Anik Ghosh, Vikas Mahajan, Abraham Peedicayil, Praveen Nath, Snita Sinukumar, Robin Thambudorai, Ramakrishnan Ayloor Seshadri, Aditi Bhatt

**Affiliations:** Department of Obstetrics & Gynecology, Command Hospital, Pune, India; Department of Anaesthesiology, Critical Care and Pain, Tata Memorial Hospital, Homi Bhabha National Institute, Mumbai, India; Department of Critical Care Medicine, Zydus Hospital, Ahmedabad, India; Department of Anaesthesiology, Cancer Institute (WIA), Chennai, India; Department of Anaesthesiology, Apollo Hospital, Chennai, India; Department of Anaesthesiology, Saifee Hospital, Mumbai, India; Bhabha Atomic Research Centre (BARC) Hospital, Mumbai, India; Department of Anaesthesiology, Jehangir Hospital, Pune, India; Department of Gynecologic Oncology, Tata Medical Centre, Kolkata, India; Department of Surgical Oncology, Apollo Hospital, Chennai, India; Department of Gynecologic Oncology, Christian Medical College, Vellore, India; Department of Anaesthesiology, Kumaran Hospital, Chennai, India; Department of Surgical Oncology, Jehangir Hospital, Pune, India; Department of Surgical Oncology, Tata Medical Centre, Kolkata, India; Department of Surgical Oncology, Cancer Institute (WIA), Chennai, India; Department of Surgical Oncology, Zydus Hospital, Ahmedabad, India

**Keywords:** cytoreductive surgery, enhanced recovery after surgery (ERAS), hyperthermic intraperitoneal chemotherapy (HIPEC), perioperative management, peritoneal metastases

## Abstract

**Objectives:**

Enhanced recovery after surgery (ERAS) protocols have been questioned in patients undergoing cytoreductive surgery (CRS) with/without hyperthermic intraperitoneal chemotherapy (HIPEC) for peritoneal malignancies. This survey was performed to study clinicians’ practice about ERAS in patients undergoing CRS-HIPEC.

**Methods:**

An online survey, comprising 76 questions on elements of prehabilitation (n=11), preoperative (n=8), intraoperative (n=16) and postoperative (n=32) management, was conducted. The respondents included surgeons, anesthesiologists, and critical care specialists.

**Results:**

The response rate was 66% (136/206 clinicians contacted). Ninety-one percent of respondents reported implementing ERAS practices. There was encouraging adherence to implement the prehabilitation (76–95%), preoperative (50–94%), and intraoperative (55–90%) ERAS practices. Mechanical bowel preparation was being used by 84.5%. Intra-abdominal drains usage was 94.7%, intercostal drains by 77.9% respondents. Nasogastric drainage was used by 84% of practitioners. The average hospital stay was 10 days as reported by 50% of respondents. A working protocol and ERAS checklist have been designed, based on the results of our study, following recent ERAS-CRS-HIPEC guidelines. This protocol will be prospectively validated.

**Conclusions:**

Most respondents were implementing ERAS practices for patients undergoing CRS-HIPEC, though as an extrapolation of colorectal and gynecological guidelines. The adoption of postoperative practices was relatively low compared to other perioperative practices.

## Introduction

Cytoreductive surgery (CRS) with or without hyperthermic intraperitoneal chemotherapy (HIPEC) has been associated with higher morbidity compared to other gastrointestinal oncological procedures, with complications developing up to 90-days after surgery [1, 2]. CRS comprises a spectrum of procedures that can vary from resection of a single peritoneal nodule to complete removal of the parietal peritoneum combined with multiple visceral resections [3]. The metabolic and inflammatory responses to surgery are greatly enhanced after such procedures [4]. Incorporation of enhanced recovery after surgery (ERAS) protocols in the perioperative management of patients undergoing CRS-HIPEC has the potential to cause an early reversal of the pathophysiological cascade and thereby hasten recovery, reduce complications and cost. In recent times, ERAS protocols have shown reproducible benefits in patients undergoing colorectal and gynecological surgeries, and guidelines have been made and are being implemented for major abdominal and extra-abdominal surgical procedures [5], [6], [7], [8], [9], [10].

Elias et al. described the normal postoperative course of patients undergoing CRS-HIPEC [11]. Events like higher pain scores, higher nasogastric tube output, higher peritoneal drain output, and transient diarrhea are often seen after CRS-HIPEC [9, 11], [12], [13]. These patients require longer intensive care admissions and a longer hospital stay, compared to other gastrointestinal surgical procedures. Webb et al. demonstrated a reduction in overall intravenous fluids, postoperative narcotic use, complication rates, and length of stay, after implementation of ERAS protocols in CRS-HIPEC patients [14]. ERAS pathways also provide an opportunity to standardize perioperative practices. An American study from 12 academic institutions showed that variation in perioperative practice patterns existed among measured ERAS pathway process/outcomes. The percentages of variation with each process/outcome measure attributable solely to institutional practices ranged from 0.6 to 66.6% [15]. We conducted this survey to study existing practices about ERAS in patients undergoing CRS-HIPEC in India.

## Materials and methods

A group of specialists comprising surgeons, anesthesiologists, and critical care specialists from the members of the Indian Network for Development of Peritoneal Surface Oncology (INDEPSO^®^) and the Society of Onco-anesthesia and Peri-operative Care (SOAPC^®^), constituted the authors of this manuscript. The initiative was taken by a core group of three members comprising a peritoneal oncology specialist (AB), one clinician experienced in perioperative management (SLS), and the third having experience with ERAS protocols and surveys (GB). The survey was then sent to the remaining authors who vetted the questionnaire considering the relevance of the questions and their choices, clarity, detail, and lack of ambiguity. The questionnaire was designed based on existing ERAS guidelines on colorectal and gynecological cancer surgery and comprised both open-ended and close-ended questions. ERAS guidelines specific to CRS-HIPEC were not available at the time this survey was conceptualized and were published subsequently [16, 17]. Several perioperative aspects specific to CRS-HIPEC and ERAS pathways were incorporated in the questionnaire (Supplementary Material 1). The online survey was designed using the Survey Monkey^®^ (SVMK, San Mateo, CA, USA) platform. Ethical approval was obtained from the Zydus Hospital Ethics Committee on July 27, 2020. The survey was open from 20th July 2020 to 16th Aug 2020. It took an average of 20 min to answer this 76-question questionnaire. Informed consent was obtained by voluntary participation from respondents. Weekly reminders were sent to those who had not answered the survey. More than 50% of the questionnaire answered was taken as a completed survey. The target audience was Indian clinicians, involved in CRS-HIPEC procedures (surgeons, anesthesiologists, and Intensivists). Electronic mails were used to disburse the survey.

The questionnaire comprised of 76 questions on clinicians’ background and experience (n=9), prehabilitation (n=11), preoperative (n=8), intraoperative (n=16), and postoperative management (n=32) of patients undergoing CRS-HIPEC. The preoperative component comprised pre-admission counseling, nutritional intervention, optimization of co-morbid conditions, correction of anemia, initiation of thromboprophylaxis, avoidance of mechanical bowel preparation (MBP) and prolonged fasting, and preoperative carbohydrate loading. Intraoperative elements included fluid and electrolyte management, specific anesthesiology practices like thoracic epidural analgesia, use of short-acting anesthetic agents and opioids, normothermia maintenance, and avoiding the use of drains. Postoperative elements included analgesia with epidural or non-opioid analgesics/short-acting opioids, prevention of nausea and vomiting, early initiation of a regular diet, early active ambulation, early removal of the drains, and oro/nasogastric tubes, thromboprophylaxis, and early discharge from the hospital.

Data from the survey were extracted in a comma-separated value (CSV) format. Statistical analysis was performed using Statistical Package for Social Sciences version 24 (SPSS 24, IBM Inc. Chicago IL, U.S.) and Microsoft Office Excel 2016 for Windows (Microsoft, Redmond, WA). Values were expressed in absolute numbers as well as percentages. The chi-square test of significance and Fisher’s exact test were used to compare differences between the responses of clinicians working with a formal institutional protocol with ERAS elements compared to those working without one. ERAS-CRS-HIPEC guidelines were available at the time this survey was analyzed. We performed a comparative subgroup analysis to assess the ‘level of agreement’ of responses with the consensus guidelines. The responses of this survey and the recently published ERAS guidelines were used by the authors to develop a working protocol and checklist, which will be prospectively incorporated in authors’ institutes. The practices that had greater than 70% agreement in the ERAS guidelines and this survey are proposed as ‘essential practices’ (elements) and those that had less than 70% concordance as ‘non-essential elements’, in the working protocol.

## Results

A total of 136 out of 206 clinicians contacted answered the survey, generating a 66% response rate. Respondents were surgical oncologists (38.2%), anesthesiologists (29.4%), gynecological oncologists (26.4%), and Intensivists (4.4%). CRS-HIPEC experience of five years or more was reported by 39.7% of respondents, and 60% reported an experience of less than five years. The majority (77.8%) of respondents were affiliated with academic institutions. The majority (95%) of respondents agreed that ERAS could be implemented in CRS-HIPEC patient population. About a third (38.2%) of respondents reported an existing ERAS protocol in their institution (Supplementary Material 2).

### Prehabilitation elements

**Figure 1:**
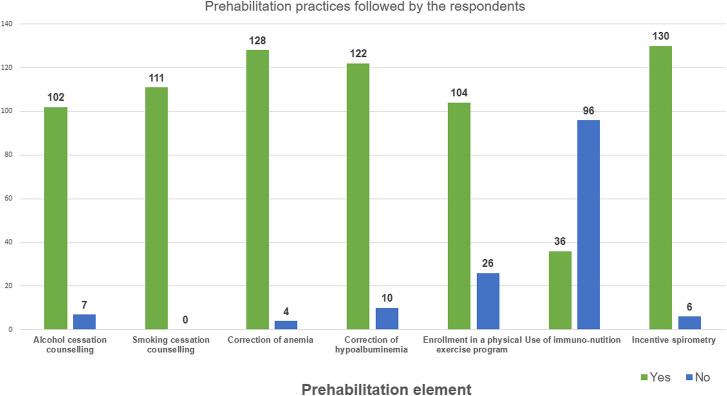
Prehabilitation practices among the respondents.

The majority of the respondent counseled patients to stop smoking (81.6%) and alcohol consumption (80.1%) before surgery, encouraged them to exercise (76.4%), and perform incentive spirometry (95%), before surgery. Preoperative anemia is corrected by most (94%) practitioners, and hypoalbuminemia by 89.7%. Immunonutrition is used only by 26.4% of clinicians in this study. Further details are presented in Supplementary Material 3 (Figure 1).

### Preoperative and intraoperative elements

MBP was reported used by 84.5% of respondents. Preoperative fasting up to 2 h for liquids was reported by 43.8% of respondents. Half (50.6%) respondents said that patients are kept fasting up to 6–8 h before surgery. The rest of the respondents used longer fasting periods preoperatively. Carbohydrate preloading was reportedly used by 40% of respondents. Thromboprophylaxis was initiated by 92.6% of respondents before surgery. Low molecular weight heparin was the commonest drug used (77.9%) for thromboprophylaxis (Tables 1 and 2).

**Table 1:** ERAS practices pertaining to preoperative preparation.

Survey question		Total number of responses to the question n=136 (%)	Number of responses, n (%)
Bowel preparation for CRS-HIPEC	– Always – For most patients – Rarely – Never	133 (97.7)	77 (56.6) 38 (27.9) 10 (7.3) 8 (5.8)
Type of bowel preparation^a^	– Mechanical bowel preparation – Mechanical + oral antibiotics – Oral antibiotics alone – No bowel preparation	133 (97.7)	92 (67.6) 25 (18.3) 3 (2.2) 13 (9.5)
Preoperative fasting for liquids	– For 2 h before surgery – For 2–6 h before surgery – For 6–12 h before surgery – For 12–24 h before surgery	133 (97.7)	59 (43.8) 42 (30.8) 28 (20.5) 4 (2.9)
Preoperative fasting for solids	– For 6 h before surgery – For 6–8 h before surgery – For 8–12 h before surgery – For 12–24 h before surgery – For >24 h before surgery	129 (94.8)	25 (18.3) 44 (32.3) 37 (27.2) 18 (13.2) 5 (3.6)
Use of carbohydrate preloading	– For all patients – For non-diabetics only – Don’t practice	134 (98.5)	18 (13.2) 37 (27.2) 79 (58.0)
Type of carbohydrate loading	– Appy juice – Glucose + water solution – Commercial ‘Pre-Carb’ preparations	87 (63.9)	36 (26.4) 31 (22.7) 20 (14.7)
Preoperative thromboprophylaxis	– Administered – Not administered	133 (97.7)	126 (92.6) 7 (5.1)
Preoperative–Intraoperative agents used for thromboprophylaxis^a^	– Low molecular weight heparin – Pneumatic compression devices – Compression stockings – Unfractionated heparin	NA	106 (77.9) 100 (73.5) 93 (67.6) 7 (5.1)

^a^Respondents could choose more than one of the options, the total may thus exceed 100%. NA, not applicable.

**Table 2:** ERAS practices pertaining to intraoperative elements.

Survey question		Total number of responses to the question n=136 (%)	Number of responses, n (%)
Multi-modal analgesia approach	– Yes – No	132 (97.0)	129 (94.8) 3 (2.2)
Components of multi-modal analgesia^a^	– Epidural + opioids + NSAIDs – Epidural + NSAIDs – TAP block + opioids – Opioids + NSAIDs	NA	104 (76.4) 35 (25.7) 18 (13.2) 10 (7.3)
Measures to reduce the use of opioids	– Yes – No	127 (93.3)	62 (45.5) 65 (47.7)
Intraoperative fluid management protocol	– Early, goal-directed therapy (non-invasive cardiac output monitoring) – Early goal-directed therapy (invasive monitoring + oesophageal Doppler study) – At the discretion of the anesthesia team – Not sure – Not present	133 (97.7)	70 (51.4) 5 (3.6) 48 (35.2) 2 (1.4) 9 (6.6)
Rate of fluid infusion during CRS phase (standard therapy)	– <2 mL/kg/h – 2–5 mL/kg/h – 5–10 mL/kg/h – >10 mL/kg/h – Other rate	128 (94.1)	12 (8.8) 46 (33.8) 41 (30.1) 16 (11.7) 13 (9.5)
Rate of fluid infusion during HIPEC phase (standard therapy)	– 10 mL/kg/h – 10–12 mL/kg/h – 12–15 mL/kg/h – >15 mL/kg/h – Other rates	123 (90.4)	29 (21.3) 28 (20.5) 31 (22.7) 18 (13.2) 17 (12.5)
Target urine output during the CRS phase	– >0.5 mL/kg/h – >1 mL/kg/h – >2 mL/kg/h – Other rates	129 (94.8)	54 (39.7) 61 (44.8) 12 (8.8) 2 (1.4)
Target urine output during the HIPEC phase	– >0.5 mL/kg/h – >1 mL/kg/h – >2 mL/kg/h – Other rates	136 (100.0)	15 (11.2) 55 (40.4) 56 (41.7) 10 (7.3)
Protocol for monitoring arterial blood gases and lactates	– At regular intervals during the CRS phase and more frequently during the HIPEC phase – At the beginning of the procedure and at fixed intervals during the CRS and HIPEC phases both – At the beginning of the procedure and at half-hourly intervals during the HIPEC phase – Others	131 (96.3)	38 (27.9) 68 (50.0) 10 (7.3) 15 (11.0)
Core temperature measurement	– Yes – No	132 (97.0)	123 (90.4) 9 (6.6)
Method of measurement of core body temperature	– Nasopharyngeal temperature probe – Oesophageal temperature probe – Tympanic membrane probe – Not measured	132 (97.0)	64 (47.0) 55 (40.4) 2 (1.4) 11 (8.0)
Methods of prevention of hypothermia^a^	– Warm intravenous fluids – Forced air blanket devices – Under-body warming mattress	NA	109 (80.1) 105 (77.2) 74 (54.4)
Measures for temperature control during HIPEC	– Stop warmer before commencing HIPEC – Cold fluids during HIPEC phase – Ice packs over the neck and axilla – Cool-air blanket	NA	111 (81.6) 89 (65.4) 56 (41.1) 38 (27.9)
Is intra-operative transfusion of packed red blood cells done routinely	– Yes – No	129 (94.8)	55 (40.4) 72 (52.9)
Hemoglobin cut-off for blood/packed red blood cells transfusion	– 7 g% – 8 g% – 9 g% – 10 g% – Other	132 (97.0)	30 (22.5) 59 (43.3) 20 (8.0) 11 (8.8) 12 (8.8)
Parameters used to decide the quantity of blood transfused^a^	– Estimated blood loss – Hemodynamic status – Intra-operative hemoglobin level – Preoperative hemoglobin level – Standard protocol	NA	10 (80.8) 87 (63.9) 82 (60.2) 51 (37.5) 5 (3.6)

^a^Respondents could choose more than one of the options, the total may thus exceed 100%. NA, not applicable.

Multimodal analgesia was used by most (94.8%) respondents. Thoracic epidurals, opioids, and non-steroidal anti-inflammatory drugs were the commonest combination (76.4%) used for multimodal analgesia. Goal-directed fluid therapy was reportedly used by 55% of respondents. Most (86%) respondents reported specific intravenous fluid infusion rates and target urine output during CRS and HIPEC phases, each (Table 2). Core body temperature was monitored by 90.4% of respondents. The majority (80%) respondents reported active measures to maintain normothermia during CRS-HIPEC procedures.

### Postoperative elements

Intensive care unit (ICU) admissions were reported by 83.8% of respondents for postoperative care. The majority (83%) reported that patients were not extubated immediately post-surgery, and were ventilated electively. Non-invasive ventilation was used by most (71%) respondents during the period of ventilation. The average length of ICU stay for patients was reported as two days by 49.2%, and four days by 30.8% of respondents. Intravenous fluids were stopped by most (82.3%) when the patient started accepting orally (Table 3).

**Table 3:** ERAS practices pertaining to post-operative management.^b^

Survey question		Total number of responses to the question n=136 (%)	Number of responses, n (%)
Immediate postoperative management			
Management in the immediate postoperative period	– Intensive care unit – High dependency unit – Surgical ward	130 (95.5)	114 (83.8) 12 (8.8) 4 (2.9)
Coagulation monitoring intra and postoperatively^a^	– PT-INR – Fibrinogen levels – Thromboelastography	NA	123 (90.4) 37 (27.2) 23 (16.9)
Post-operative ventilation	– Ventilate select patients – Ventilate all patients – Extubate all patients on table – Other responses	134 (98.5)	87 (63.9) 26 (19.1) 14 (10.2) 7 (5.1)
Use of non-invasive ventilation (NIV) in the post-operative period	– For some – For all – Don’t use	134 (98.5)	81 (59.5) 16 (11.6) 37 (27.2)
Duration of non-invasive ventilation (NIV)	– 3 days – 5 days – As long as clinically indicated – During ICU stay – Other responses	123 (90.4)	8 (5.8) 7 (5.1) 65 (47.7) 31 (22.7) 12 (8.8)
Average length of stay in the intensive care unit	– 1 day – 2 days – 4 days – 7 days – Other responses – Missing	133 (97.7)	12 (8.8) 67 (49.2) 42 (30.8) 5 (3.6) 7 (5.1) 3 (2.2)
Fluid and electrolyte balance, diuretics			
Rate of fluids administration in the post-operative period	– <40 mL/h – 40–100 mL/h – >100 mL/h	125 (91.9)	3 (2.2) 56 (41.1) 65 (47.7)
Use of intravenous fluids	– 12–24 h after surgery – More than 24 h after surgery – Depends on the clinical condition (when oral feeds are tolerated)	134 (98.5)	6 (4.4) 16 (11.7) 112 (82.3)
Frequency of serum calcium, magnesium and phosphate levels monitoring	– As clinically indicated – Daily – Alternate day – Other responses	131 (96.3)	58 (42.6) 46 (33.8) 22 (16.1) 5 (3.6)
Use of diuretics	– Not used – Yes, if clinically indicated – Yes for all	133 (97.7)	68 (50.0) 62 (45.5) 3 (2.2)
Standard protocol for post-operative hypotension, post-HIPEC	– Yes – No	127 (93.3)	65 (47.7) 62 (45.5)
Threshold for adding vasopressor/inotropic support to fluid resuscitation for non-responders^a^	– Adequate fluid resuscitation done – Evidence of end-organ hypoperfusion (e.g., cardiac symptoms, renal failure, confusion, etc.) – Signs of volume overload (pulmonary oedema, pleural effusions) – Cardiac output numbers	NA	95 (69.8) 64 (47.5) 30 (22.0) 31 (22.7)
Perioperative nutritional care			
Commencement of pre-emptive enteral feeding	– <24 h after surgery – 24–48 h after surgery – >48 h after surgery – Other responses	133 (97.7)	12 (8.8) 62 (45.5) 47 (34.5) 12 (8.8)
Use of pre-emptive parenteral nutrition	– Always – Sometimes – Rarely – Never	132 (97.0)	36 (26.4) 60 (44.0) 30 (22.0) 6 (4.4)
Agents used to hasten return of bowel function^a^	– No agents used – Prokinetic drugs (Cispride/Mosapride) – Bisacodyl suppositories – Chewing gum – Milk of magnesia – Erythromycin	NA	71 (52.2) 37 (27.2) 23 (16.9) 19 (13.9) 7 (5.1) 4 (2.9)
Established protocol for prevention/management of postoperative nausea-vomiting	– Yes – No	134 (98.5)	97 (71.3) 37 (27.2)
Drugs used for prevention/management of postoperative nausea-vomiting^a^	– Metoclopramide – H_2_ receptor antagonists – Dexamethasone – Aprepitant – Others	NA	66 (48.5) 63 (46.3) 50 (36.7) 15 (11.0) 2 (1.4)
Commencement of a regular diet	– 24–48 h after surgery – 48–72 h after surgery – >72 h after surgery – Depends on the extend of surgery, number and site of bowel anastomosis	131 (96.3)	5 (3.6) 16 (11.7) 26 (19.11) 84 (61.7)
Removal of drains, nasogastric tube, urinary catheter			
Use of intra-abdominal drains	– Always – Sometimes – Rarely – Never	133 (97.7)	112 (82.3) 17 (12.5) 4 (2.9) 0 (0.0)
Removal of drains	– Post-operative day 1 – Post-operative day 3 – Post-operative day 5 – Post-operative day 7 – When the desirable drain output is reached (or reduces below a pre-specified level)	128 (94.1)	2 (1.4) 20 (14.7) 15 (11.2) 2 (1.4) 89 (65.4)
Use of inter-costal drains	– For all patients undergoing diaphragmatic peritonectomy – Only if diaphragmatic resection – Rarely used – For all HIPEC procedures – Other responses	129 (94.8)	54 (39.7) 52 (38.2) 17 (12.5) 4 (2.9) 2 (1.4)
Average number of drains (including thoracic), if used	– 1 – 2 – 3 – 4 – Other responses	128 (94.8)	14 (10.2) 40 (29.2) 32 (23.5) 28 (20.5) 14 (10.2)
Urinary catheter removal after CRS-HIPEC	– 24–48 h after surgery – 48–72 h after surgery – >72 h after surgery – When the patient becomes ambulant – Other responses	131 (96.3)	11 (8.0) 33 (24.2) 29 (21.3) 50 (36.7) 8 (5.8)
Post-operative use of a nasogastric tube	– Always – Sometimes – Rarely – Never	134 (98.5)	104 (76.4) 24 (17.6) 4 (2.9) 2 (1.4)
Removal of the nasogastric tube	– 24 h after surgery – 48 h after surgery – 72 h after surgery – When oral feeds are tolerated – When the output reduces/bowel function returns – Other responses	131 (96.3)	27 (19.8) 21 (15.4) 5 (3.6) 31 (22.7) 42 (30.8) 5 (3.6)
Thromboprophylaxis, mobilization and discharge			
Pharmacological thromboprophylaxis	– Yes – No	133 (97.7)	132 (97.0) 1 (0.7)
Duration of thromboprophylaxis	– For 2 weeks after surgery – For 4 weeks after surgery – During the hospital stay alone – During the ICU stay alone – Other responses	130 (95.5)	18 (13.2) 20 (14.77) 70 (51.4) 12 (8.8) 10 (7.3)
Commencement of mobilization	– One the day of surgery – Post-operative day 1 – Post-operative day 2 – Post-operative day 3 – Other responses	131 (96.3)	1 (0.7) 69 (50.7) 46 (33.8) 12 (8.8) 3 (2.2)
Average hospital stay	– <7 days – 7–10 days – 10–12 days – >12 days	130 (95.5)	17 (12.5) 61 (44.8) 41 (30.1) 11 (8.0)

^a^Respondents could choose more than one of the options, the total may thus exceed 100%. ^b^Responses to three questions are not listed in this table (1 with subjective responses, 2 on ERAS protocols). NA, not applicable.

Most (94.7%) respondents used intraabdominal drains. These drains were removed when the output reduced below a subjectively determined volume (65.4% respondents). Most (77.9%) reported using intercostal drains when diaphragmatic surgery/peritonectomy was performed. Urinary catheters were kept up to 72 h by 32.2% of respondents, 36.7% removed these when the patient became ambulant. The majority (84%) used nasogastric tubes in the postoperative period. Thromboprophylaxis was continued in the postoperative period by most (97%) respondents. The average hospital stay was reported between seven to 10 days by 44.8% of respondents, while 10–12 days was reported by another 30%.

There were some differences in the practices among clinicians working with and without a formal protocol incorporating ERAS practices. The only statistically significant one was the use of preoperative carbohydrate loading that was more in the ERAS group (p=0.002) (Table 4). The ‘commonest choices among responses’ for ERAS elements were compared with the consensus guidelines. The percentage of agreement was low (50–75%) for postoperative practices like avoiding nasogastric drainage, intra-abdominal drains, and thoracic drains, which was similar to the practices of our respondents (Table 4).

**Table 4:** Comparison of responses from clinicians with and without institutional protocols with ERAS elements^a^ and comparison with the responses of specialists participating in the ERAS consensus guidelines.

Survey question	Most common response	Existing ERAS protocol, n (%)	No ERAS protocol, n (%)	All patients, n (%)	p-Value^b^	ERAS consensus guidelines (level of agreement (%) amongst the specialists)
Do you use bowel preparation before CRS and HIPEC	Always	52 (64.1%)	25 (48.0%)	77 (58.7%)	0.225	62.5% (for rectal resection) 54.2% (not required for colonic resection)
Do you use carbohydrate loading in your preoperative practice?	I don’t use carbohydrate loading	21 (40.3%)	56 (70.05%)	77 (58.3%)	0.002	Preoperative carbohydrate loading should be used (75.0%)
When is a regular diet started routinely after CRS-HIPEC?	Depends on the extent of surgery, number and site of bowel anastomosis	49 (63.6%)	34 (65.3%)	83 (64.3%)	0.872	Solid food should be started on postoperative day 1 (66.5%)
What is your practice for using intra-abdominal drains?	Always	69 (86.2)	42 (80.7)	111(84.0)	0.3192	Intra-abdominal drains should be used (50.0%)
What is your practice for using inter-costal drains?	For all patients undergoing diaphragmatic peritonectomy	29 (37.6)	25 (48.0)	54 (41.8)	0.701	Thoracic drainage should be performed for patients undergoing diaphragmatic surgery (54.2%)
When is the urinary catheter commonly removed after CRS and HIPEC	When the patient starts ambulating	28 (35.4)	21 (41.1)	49 (37.6)	0.865	Urinary catheter should be removed after 3 days (83.3%)
Do you retain a nasogastric tube post-operatively?	Always	62 (77.5)	40 (79.6)	102 (77.2)	0.325	Nasogastric tube should not be retained in absence of risk factors for delayed gastric emptying (54.2%)
For how long is postoperative thromboprophylaxis continued?	During hospital stay only	38 (50.0)	30 (57.6)	68 (53.1)	0.174	Thromboprophylaxis should be continued for 4 weeks post-operatively (95.8%)

^a^Only responses to selected questions have been compared here. ^b^Comparison between clinician working with and without a formal ERAS protocol.

### Working protocol and checklist

**Figure 2:**
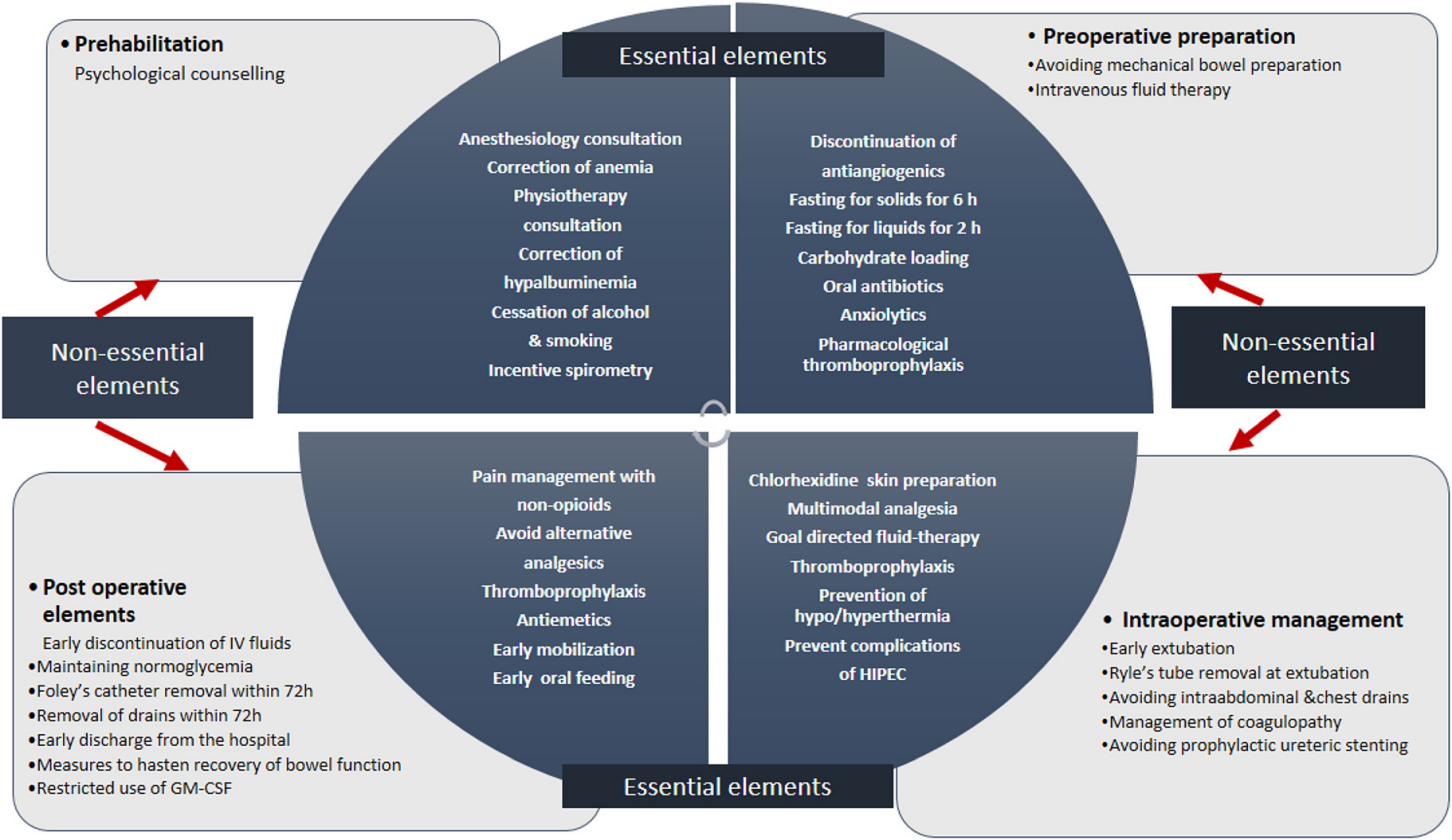
Essential and non-essential elements in the working protocol for prospective implementation of ERAS practices.

The working protocol (Supplementary Material 4) includes 44 elements that are divided into essential and non-essential elements (Figure 2). Essential elements are those that must be followed in all patients irrespective of the disease extent and nature of surgery. Non-essential elements are those which can be selectively implemented at the discretion of the treating clinicians.

## Discussion

This survey, addressing ERAS practices about CRS-HIPEC, showed that the majority (91%) of the clinicians reported implementing ERAS practices. Anesthesiologists and critical care specialists who form an integral part of the team managing CRS-HIPEC patients were part of this study. This adds value to this study since they form one of the ‘ERAS champions’ along with surgeons and nurses [18]. Similar surveys in past [19], [20], [21] had invited only surgeons to respond. ERAS practices are gradually being incorporated as institutional protocols. Our study showed that 38% of respondents already have ERAS practices instituted in their protocols. This aligns with a recent international survey among gynecological oncology surgeons [21]. It was encouraging to see that most (91%) other institutes were following some of the ERAS practices, more importantly, as no formal ERAS CRS-HIPEC guidelines were available at the time of this study. Peritoneal surface malignancy is an evolving surgical discipline and is a decade old in India [22]. Most clinicians involved in CRS-HIPEC practice for more than 5 years in India answered this survey.

### Prehabilitation

Benefits of prehabilitation have been shown in several recent studies, and these form integral initial elements in an ERAS pathway [23], [24], [25], [26]. It was encouraging to see that most clinicians were following prehabilitation practices in their CRS-HIPEC subset of patients (75–95%). Incentive spirometry was initiated by most (95%) when a decision of surgery was made [27]. Our previous report showed a reduction in respiratory complications in patients undergoing CRS-HIPEC with preoperative spirometry and non-invasive ventilation in the postoperative period [28, 29].

### Preoperative elements

The current consensus is to avoid mechanical bowel preparation for major abdominal and pelvic surgeries except for rectal surgeries, though bowel preparation with oral antibiotics is recommended [30]. The majority (86%) of respondents still use bowel preparation for their CRS-HIPEC procedures. In the international consensus, 65.2% recommended MBP for patients undergoing rectal resection, which is low considering that these patients have higher rates of bowel resections and multiple anastomoses. Most of our patients present with a high peritoneal cancer index, that merits multiple bowel resections, and most commonly, a rectal resection [28, 31]. There are two theoretical benefits of bowel preparation – reduction in leak rates and wound infections. Anastomotic leaks after CRS-HIPEC are difficult to manage [32, 33]. This and the possibility of unplanned rectal resections are likely reasons for a more conservative approach. The side-effects of mechanical bowel preparation are patient discomfort, dehydration, and prolonged hospital stay [17]. ‘Modern fasting’ practices were reported as 43.8% for liquids (2 h before surgery) and 50.6% for solids (6–8 h before surgery), respectively [34]. There is still a large scope of improvement in these elements. More robust clinical evidence is needed before avoiding bowel preparation can be systematically incorporated into ERAS practices.

### Intraoperative elements

Forty-five% respondents reported that they were actively trying to reduce opioid use, usually by alternative regional analgesia techniques like transversus abdominis plane block, wound infiltration, etc. Similarly, the use of epidural analgesia, which has shown advantages for other surgical procedures, was high (>75.0%) [18].

In the recently published ERAS guidelines, there are many other components like measures for preventing surgical site infections, skin preparation, maintenance of normothermia, optimal fluid management using goal-directed fluid therapy that is already part of the perioperative management protocols [14, 17, 35]. Majority of the respondents followed these practices. The SOAPC has published consensus guidelines for the perioperative management of patients undergoing CRS-HIPEC that already address most of these issues [36]. Some specific measures were not included in our survey questionnaire as they represent routine practices like skin preparation and prophylactic oral antibiotics.

### Postoperative management

More than 50% of the respondents favored early extubation, if possible, in the operating room, early mobilization, and early initiation of feeding, which concurs with ERAS practices. But extubation in the operating room or elective ventilation depends on many factors like preoperative pulmonary and cardiac comorbidities, resection of the diaphragm, amount of blood loss during surgery, and hemodynamic stability at the end of surgery [37]. The respondents’ approach was more conservative regarding other practices like avoiding nasogastric drainage or early removal, avoiding/early removal of intraabdominal and thoracic drains, and early removal of the urinary catheter. Once again, this could be attributed to the extent of the surgery. It’s been proposed that nasogastric drainage can be avoided in patients who do not undergo resection of the lesser omentum [14]. In our protocol, these practices are optional, and we will prospectively evaluate the results of these practices using the checklist. Another essential aspect that is difficult to replicate in the Indian subcontinent is early discharge [35]. When the cost is borne by patients directly, both clinicians and patients are more comfortable with a few extra days in the hospital than early discharge, which is less than the cost of taking essential supportive care at another medical facility. Patients coming from smaller towns and remote areas do not have access to the supportive care required following these procedures (supportive fluid therapy, drainage of collections). Traveling back and forth is expensive and inconvenient. The postoperative practices are still conservative and need the maximum transition to ERAS pathways. It has been shown that out of several ERAS elements, postoperative elements have the greatest impact on optimal recovery [37]. However few recent studies have demonstrated that postoperative elements are the most difficult ones to implement [38, 39].

### Implementation of ERAS protocol and checking compliance

The working protocol comprises essential and non-essential elements (Figure 2). Compliance of 70% is acceptable when evaluating adherence to ERAS protocols. But this may not be applicable for all elements. Some elements like prehabilitation, shorter duration of fasting, carbohydrate loading, multimodal analgesia, and early ambulation can be implemented in all patients, irrespective of disease extent and surgical complexity (Supplementary Material 4), and the compliance for such elements should be nearly 100%. Implementation of these can lead to a reduction in morbidity and length of stay. Others like avoiding MBP, early removal of drains, and early discharge may be difficult to implement in all patients and more evidence is needed to recommend their routine use. Studies reporting the feasibility of ERAS in patients undergoing CRS have also demonstrated lower compliance for the latter [35, 39]. This division into essential and non-essential elements should make it feasible for more teams to follow these practices. The accompanying checklist is a simple and inexpensive tool to both document and evaluates compliance.

### Future directives

Implementation of the protocol and check-list will enable us to devise a final protocol that can be implemented with a high level of compliance at our centers. It will be used to analyze the impact of these practices on morbidity and mortality. Based on the results and accumulating evidence from around the world we hope to be able to create an ‘ideal’ protocol for the Indian centers. The results will also be discussed at clinical meetings and conferences to recruit more centers for the study. Members of both societies will be informed thorough emails and social media platforms about the outcomes of the prospective study. The results will also be published in scientific journals.

Our survey was exhaustive considering ERAS practices alone and had a reasonable response rate (>60%). Nearly 100% of the surgeons exclusively involved in the management of peritoneal metastases responded to the survey. This survey serves as a baseline evaluation of the existing practices. The working protocol and checklist can be used by other teams to implement ERAS practices. There are certain weaknesses of our study. Firstly, it is a survey-based study, thus represents clinicians’ opinions. There is always a possible gap between responses and actual practice in a survey-based study. The strengths of our study are that this survey represents the perspectives of anesthesiologists and Intensivists too. Prior surveys have not engaged these practitioners. Our survey is possibly the most exhaustive survey yet on CRS-HIPEC, with 76 questions. We feel this study captures most perioperative elements involved in the CRS-HIPEC subset of patients. The prospective validation of ERAS-CRS-HIPEC guidelines will soon be undertaken, and we would be able to contribute to ever-emerging evidence supporting ERAS.

## Conclusions

This national survey demonstrates that the majority of the respondents reported incorporating ERAS practices for patients undergoing CRS-HIPEC. Many of the preoperative and intraoperative practices were being followed. The adoption of several postoperative practices was relatively lower. There is institutional variation in several perioperative practices, and ERAS protocols provide an opportunity to streamline these variations. The working protocol and checklist will formalize the implementation of these practices and evaluate the clinical benefit and safety of ERAS practices.
