# Supplementary material for: Enhanced recovery after surgery (ERAS) in cytoreductive surgery (CRS) and hyperthermic intraperitoneal chemotherapy (HIPEC): a cross-sectional survey
Source: Pleura Peritoneum. 2021 Jun 21;6(3):99–111. doi: 10.1515/pp-2021-0117 (PMC8482448; doi:10.1515/pp-2021-0117)
Supplement: Supplementary file 1 [file pp-06-20210117-s001.docx]

**Supplement 4**

**ERAS practices- Working guidelines and compliance Check-list**

**Guidelines in brief**

These guidelines are for all patients undergoing cytoreductive surgery with or without hyperthermic intraperitoneal chemotherapy (HIPEC)

Implementation of the enhanced recovery after surgery (ERAS) protocol begins on the day the surgery has been scheduled/planned. There are some essential and non-essential elements

**Prehabilitation**

Waiting period for surgery (time from decision of surgery to actual date of surgery) should be utilized for a holistic approach toward prehabilitation and optimization of comorbid conditions.

1. Preadmission counselling and consultation with the anesthesiology team for optimization of comorbidities and assessment of risk should be performed for all patients.
2. Assessment of nutritional status including serum albumin and Pre-Operative Nutrition Score (PONS) score. Nutritional supplements should be given for a minimum of two weeks in patients who have a poor nutritional status
3. Blood profile with assessment of hemoglobin- anemia should be corrected with oral or intravenous iron and when indicated, packed cell transfusion
4. Physiotherapy consultation and enrolment in an exercise program. If the same is not possible, patients should be asked to start some form of physical exercise prior to surgery like a brisk walk for 30 minutes, daily
5. Cessation of alcohol and smoking- should be strongly advised to all patients
6. Incentive spirometry- should begin for all patients on the day of first consultation. If the patient is seen while still on chemotherapy, incentive spirometry should be advised during that period too.
7. Psychological counselling

**Preoperative preparation**

1. Stopping anti-angiogenic medications 4 weeks prior to surgery
2. Admission one day prior to surgery for all patients
3. Fasting for solids for 6 hours before surgery
4. Fasting for liquids for 2 hours prior to surgery
5. Carbohydrate loading till 2 hours prior to surgery (except in diabetics)
6. Oral antibiotics for bowel preparation, the day before surgery
7. Anxiolytic and antacids on the night before surgery
8. Pharmacological thromboprophylaxis 12 hours prior to surgery
9. Mechanical bowel preparation- can be avoided for patients not requiring rectal resection
10. Intravenous fluid therapy- can be considered in patients with compromised renal function and/or those receiving Cisplatin HIPEC

**Intraoperative measures**

1. Skin preparation with Chlorhexidine
2. Multimodal analgesia – One or more of the following: thoracic epidural catheter where no contraindications exist; transversus abdominis block; local anesthetic infiltration
3. Goal directed fluid therapy to prevent volume overload as well as tissue hypoperfusion.
4. Mechanical thromboprophylaxis during surgery
5. Active measures for prevention of hypo/hyperthermia
6. Sodium thiosulfate or amifostine to be used when high-dose (>75mg/m2) of Cisplatin is used for HIPEC
7. Avoid use of very high dose of Cisplatin (>240mg) in all patients and Mitomycin C>40mg in patients with risk factors
8. Early extubation
9. Avoiding post-operative nasogastric drainage
10. Avoiding intraabdominal drains
11. Avoiding chest drains
12. Avoid prophylactic ureteric stenting
13. Management of coagulopathy

**Post-operative measures**

1. Pain management with non-opioids and selective use of opioids
2. Avoid the use of alternative analgesics (ketamine, lidocaine and gabapentin)
3. Thromboprophylaxis- mechanical and pharmacological during hospital stay
4. Antiemetics for preventing post-operative nausea and vomiting (PONV)
5. Early mobilization on day0/1 of extubation
6. Early initiation of oral feeding and selective use of preemptive enteral and parenteral feeding
7. Early discontinuation of intravenous fluids
8. Glucose monitoring and use of short-acting insulin to maintain normoglycemia
9. Removal of Foley’s catheter within 72 hours of surgery
10. Removal of intra-abdominal drains within 72 hours of surgery
11. Removal of thoracic drains within 72 hours of surgery
12. Early discharge from the hospital
13. Measures to hasten the recovery of bowel function
14. Restricted use of granulocyte monocyte colony stimulating factor (GM-CSF)

ERAS checklist for compliance– this should be filled for every patient.

Table 1- Clinical and surgical details

| First name initial ___ Surname initial _____  Age(yrs) ____ Sex: M ___ F ___ BMI_______  Centre ______________ City __________ Country _____________  Treating clinician ________________ |
| --- |
| Primary tumor site ________ Date of first diagnosis ___________  Date of diagnosis of PM _________ |
| Date of first consultation for surgery _______________  Date of surgery _________________  PCI _______ CC-score ______________  Peritonectomy procedures: Total Number ____  Right upper quadrant __ Left upper quadrant ____ Pelvic ____  Anteroparietal: right _____ left______  Greater omentectomy _____ Lesser omentectomy ______  Mesenteric peritonectomy Total _____ Regional/partial ______ Focal resection _____-  Electro-evaporation of mesenteric nodules _______  Visceral resections  No of visceral resections ______  List the resected viscera _______________________________________  Diaphragm resection: Right ___ Diameter _____ Left ______ Diameter_____  Lymph node dissection (list regions) __________________________  HIPEC Yes__ No___ Duration ____(mins) Drug/s ________________  Number of bowel anastomosis ______  Rectal anastomosis _______  Diverting o’stomy: Site ___________ Temporary______ Permanent ________ |

Table 2 ERAS Check-list (please tick one or more options in columns 2 and 3). Any other interventions can be listed in the comments.

| Clinical parameter | |  | Specific measure(s) for  improvement | Comments |
| --- | --- | --- | --- | --- |
| **Prehabilitation** | | | | |
|  | Cessation of alcohol and smoking | Smoker [_]  Alcoholic [_] | Counselled [_]  Not counselled [_] |  |
|  | Preoperative counselling | Done [_]  Not done [_] | Tools used  Written content [_]  Videos [_] |  |
|  | Optimization of co-morbidities | Done [_]  Not done [_] | Comorbid condition  Diabetes Mellitus [_]  Hypertension [_]  Chronic lung disease [_]  Ischemic heart disease [_]  Any other [_] |  |
|  | Physical exercises | Performing regularly [_]  Not performing [_] | Counselled [_]  Not counselled [_] |  |
|  | Physiotherapy consultation |  | Done [_]  Not done [_]  Not required [_] |  |
|  | Incentive spirometry |  | Advised [_]  Not advised [_] |  |
|  | Anemia correction  Required if Hb<11.0g% | Required [__]  Done [_]  Not done [_] | Oral Iron [_]  Parenteral Iron [_]  Transfusion [_]  Vit B12 [_] |  |
|  | Albumin level (<3.0g/dl is considered low) | Normal [_]  Low [_] | High protein diet [_]  Dietician consultation [_]  Structured proteins [_]  Albumin transfusion [_] |  |
|  | Immuno-nutrition | Advised [_]  Not advised [_] | Duration _______ (days) |  |
|  | PONS Score (Preoperative nutrition score) | Nutritional intervention  Required [_]  Not required [_] | Weight loss <10% in 6m [_]  BMI<18.5 [_]  Oral intake <50% of normal [_]  Serum albumin <3.0g/Dl [_] |  |
| **Pre-operative preparation** | | | | |
|  | Antiangiogenic drugs | Administered [_]  Not administered [_] | Time between last dose and surgery  4 weeks [_]  5 weeks [_]  6 weeks [_]  More than 6 weeks [_] |  |
|  | Bowel preparation | Done [_]  Not done [_] | Mechanical [_]  Oral antibiotics [_]  Enema [_] |  |
|  | Preoperative sedation | Given [_]  Not given [_] | Drugs used (list) |  |
|  | Nausea-Vomiting prophylaxis | Given [_]  Not given [_] | Anti-emetics [_]  Inhalation anesthetics  Used [_]  Not used [_] |  |
|  | Duration of preoperative fasting for solids |  | 2 hours [_]  4 hours [_]  6 hours [_]  >6 hours [_] |  |
|  | Duration of preoperative fasting for liquids |  | 2 hours [_]  4 hours [_]  6 hours [_]  >6 hours [_] |  |
|  | Preoperative carbohydrate loading | Performed [_]  Not performed [_] | Gatorade [_]  Glucose + water solution [_]  Pre-Carb [_]  Appy Juice [_] |  |
|  | Pharmacological thromboprophylaxis before surgery | Initiated [_]  Not initiated [_] | Low molecular weight heparin [_]  Unfractionated heparin [_]  Fondaparinox [_] |  |
|  | Preoperative antibiotics | Given [_]  Not given [_] | List drugs |  |
|  | Intravenous hydration | Performed [_]  Not performed [_] | <24 hours [_]  24 hours [_]  48 hours [_] |  |
| **Intra-operative management** | | | | |
|  | Antibiotic prophylaxis and skin preparation | Chlorhexidine skin preparation [_]  Antibiotic prophylaxis [_]  Additional anti-SSI measures [_] | List additional measures |  |
|  | Standard anaesthetic protocol | Rapid sequence intubation [_]  Epidural analgesia [_]  Multimodal analgesia  [_]  Protective mechanical ventilation [_]  Cardiac output monitoring [_] |  |  |
|  | Multimodal analgesia approach | Used [_]  Not used [_] | -Epidural + Opioids + NSAIDs [_]  -Epidural +NSAIDs [_]  -Opioids + NSAIDs [_]  -TAP blocks +Opioids [_] |  |
|  | Goal directed fluid-therapy (during the CRS phase) | Used [_]  Not used [_] | <2 ml/kg/hr [_]  2-5 ml/kg/hr [_]  5-10 ml/kg/hr [_]  >10 ml/kg/hr [_] |  |
|  | Goal directed fluid-therapy (during the HIPEC phase) |  | 10 ml/kg/hg [_]  10-12 ml/kg/hr [_]  12-15 ml/kg/hr [_]  >15 ml/kg/hour [_]  Any other [_] |  |
|  | Target urine-output (during the CRS phase) |  | >0.5 ml/kg/hour [_]  >1 ml/kg/hour [_]  >2ml/kg/hour [_] |  |
|  | Target urine-output (during the HIPEC phase) |  | >0.5 ml/kg/hour [_]  >1 ml/kg/hour [_]  >2ml/kg/hour [_] |  |
|  | Core body temperature monitoring | Performed [_]  Not performed [_] | Oesophageal temperature probe [_]  Nasopharyngeal temperature probe [_]  Tympanic probe [_] |  |
|  | Maintaining normothermia during CRS | Performed [_]  Not performed [_] | Forced air warmer [_]  Warm IV fluid [_]  Heated under-body blanket [_] |  |
|  | Maintaining normothermia during HIPEC | Performed [_]  Not performed [_] | Cool-Air blanket [_]  Stop Warmer before [_]  HIPEC phase starts  Using cold fluids during the HIPEC phase [_]  Ice packs over neck and axilla [_] |  |
|  | Monitoring of coagulation parameters | Performed [_]  Not performed [_] | PT/INR [_]  Thromboelastography [_]  Fibrinogen levels [_] |  |
|  | Monitoring of lactates and arterial blood gases | Performed [_]  Not performed [_] | At the beginning of the CRS phase [_]  At the beginning of the HIPEC phase [_]  Every 30 mins during the HIPEC phase [_]  As clinically indicated [_] |  |
|  | Tranfusion and management of coagulopathy | Restricted blood transfusion [_]  Preemptive use of fresh frozen plasma [_]  Tranexemic acid [_] | Blood products (mention units)  Packed red blood cells ___  Fresh frozen plasma ___  Platelet rich plasma ___  Cryoprecipitate _____  Whole blood ____  Any other _____ |  |
|  | Maintaining normoglycemia | Diabetes screening [_]  Intra-operative measures [_] | Frequency of blood glucose monitoring  2-hourly [_]  4 –hourly [_]  6-hourly [_] |  |
|  | Prevention of HIPEC related complications | Drug 1 __________  Dose _______  Drug 2__________  Dose ___________ | Loop diuretics [_]  Dopamine [_]  Sodium thiosulphate [_]  Amifostine [_]  Granulocyte colony stimulating factor [_] |  |
| Post-operative management | | | | |
|  | Immediate post-operative management | Intensive care unit [_]  High dependency unit [_]  Ward [_] | Duration ____ (days) |  |
|  | Post-operative invasive ventilation | Performed [_]  Not performed [_] | Duration ____ (hours)  If Yes, extubated on POD ___ |  |
|  | Post-operative non-invasive ventilation | Performed [_]  Not performed [_] | Continuous [_]  Intermittent [_]  Duration ______ (days) |  |
|  | Monitoring electrolytes in the immediate post-operative period | Once a day [_]  Twice a day [_]  Every alternate day [_]  As clinically indicated [_] |  |  |
|  | Antibiotic prophylaxis | No [_]  Yes [_]  Duration ____ days | List drugs |  |
|  | Mobilization | On the day of surgery [_]  First post-operative day [_]  After extubation [_]  2^nd^ POD or later [_] |  |  |
|  | Pain management | Epidural [_]  TAP block [_]  Wound infiltration [_]  Oral [_]  Intravenous [_]  Transdermal [_] | Opioids [_]  Non-opioids [_]  NSAIDs [_]  Paracetamol [_] |  |
|  | Intravenous fluid therapy | Duration _____ (days) | < 40ml/h [_]  40ml/h – 100ml/h [_]  > 100 ml/h [_]  No IV fluids given [_] |  |
|  | Nasogastric drainage | Performed [_]  Not performed [_] | <48 hours [_]  48-72 hours [_]  >72 hours [_] |  |
|  | Abdominal drains | Number of drains  0 [_]  1 [_]  2 [_]  >3 [_] | <48 hours [_]  48-72 hours [_]  >72 hours [_]  >5 days [_] |  |
|  | Intercostal drainage | Number of drains  0 [_]  1 [_]  2 [_] | <48 hours [_]  48-72 hours [_]  72-120 hours [_]  >5 days [_] |  |
|  | Urinary catheter |  | <48 hours [_]  48-72 hours [_]  72-120 hours [_]  >5 days [_] |  |
|  | Preemptive enteral feeding | Initiated [_]  Not initiated [_] | <48 hours [_]  48-72 hours [_]  >72 hours [_] |  |
|  | Oral liquids |  | <48 hours [_]  48-72 hours [_]  >72 hours [_] |  |
|  | Normal diet |  | <48 hours [_]  48-72 hours [_]  >72 hours [_] |  |
|  | Preemptive parenteral nutrition | None [_]  Amino-acids [_]  Intralipids [_]  Combination [_] | <48 hours [_]  48-72 hours [_]  >72 hours [_]  >5 days [_] |  |
|  | Diuretics | Used  Not used | Loop diuretics [_]  Potassium sparing [_]  Dopamine [_] |  |
|  | Thromboprophylaxis prophylaxis | Pharmacological [_]  Mechanical [_]  Both [_] | During ICU stay [_]  During hospital stay [_]  2 weeks [_]  4 weeks [_] |  |
|  | Hastening recovery of bowel function | Active intervention  Performed [_]  Not performed [_] | Epidural analgesia [_]  Prokinetics [_]  Milk of magnesia [_]  Bisacodyl suppositories [_]  Chewing gum [_] |  |
|  | GM-CSF | Used [_]  Not used [_]  No of doses [_] | Lowest white cell count  3000 [_]  1500-3000 [_]  500- 1500 [_]  <500 [_] |  |

Table 3 Additional information

| Hospital stay (Days)___  Readmission (Post-discharge) Yes__ No___  Reason for readmission ________________ |
| --- |
| 90-day morbidity Yes__ No __  Grade 1-2 complications ___________ Grade 3-4 complications ___________  List complications _________ |
| Adjuvant Chemo _____________ As scheduled __ Delayed ___  Delay duration & cause, if applicable _____________________________ |
